# Evaluation of Hemodynamic Changes in Retrobulbar Blood Vessels Using Color Doppler Imaging in Diabetic Patients

**DOI:** 10.3390/life12050629

**Published:** 2022-04-23

**Authors:** Gulshan Madhpuriya, Sudheer Gokhale, Alka Agrawal, Prakhar Nigam, Yung-Liang Wan

**Affiliations:** 1 Department of Radiodiagnosis, M.G.M. Medical College and M.Y. Hospital, Indore 452001, India; gkmadhpuriya@gmail.com (G.M.); alka.usg@gmail.com (A.A.); prakh3217@gmail.com (P.N.); 2 Gokhale Sonography Clinic, Indore 452001, India; drgokhale.sudheer@gmail.com; 3 Department of Medical Imaging and Intervention, Linkou Chang Gung Memorial, College of Medicine, Chang Gung University, Taoyuan City 333, Taiwan

**Keywords:** diabetes, retinopathy, orbital artery, Doppler, hemodynamic

## Abstract

Background—Diabetic retinopathy is a common complication of long-standing hyperglycemia. Microangiopathy-induced retinal changes are well-visualized on ophthalmoscopic examination. However, certain hemodynamic alterations have also been documented in the diabetic population, which have not been completely understood. Aim—To study the hemodynamic changes in retrobulbar circulation in diabetic patients with and without retinopathy, and to compare these changes with non-diabetic controls. Materials and Methods—This hospital-based prospective study included 50 diabetic and 50 non-diabetic patients. The diabetic groups consisted of 25 patients without retinopathy and 25 patients with retinopathy, and were labeled as Groups I and II, respectively. All subjects underwent orbital color Doppler ultrasonography using a linear high-frequency probe. The color Doppler parameters (peak systolic velocity (PSV), end-diastolic velocity (EDV), and resistive index (RI)) were measured and recorded using the spectral waveform of the ophthalmic artery (OA), central retinal artery (CRA), and short posterior ciliary arteries (SPCA). Comparison of obtained values was carried out using appropriate tests of significance. Results—The resistive indices of the ophthalmic, central retinal, and short posterior ciliary arteries were significantly higher in diabetic patients compared to controls (*p* < 0.001). The difference was also significant between Group I and Group II. Comparison of PSV and EDV of CRA and SPCAs among three groups using one-way ANOVA revealed a significant difference, with the highest blood flow velocities in the control group and the lowest in diabetics with retinopathy. The ophthalmic artery showed no significant change in blood flow velocity. Analysis using the Pearson correlation coefficient provided a positive correlation between the RI values of OA, CRA, and SPCA and the presence of diabetic retinopathy (OA = r 0.417, *p* < 0.001; CRA = r 0.466, *p* < 0.001; SPCA = r 0.438; *p* < 0.001). Conclusions—The resistive index of OA, CRA, and SPCA is a reliable indicator to assess diabetic-associated hemodynamic changes. The use of orbital color Doppler ultrasonography in diabetic patients can help in the identification of patients who are at risk of developing retinopathy.

## 1. Introduction

Diabetes mellitus causes a myriad of systemic complications, among which ocular complications are a major threat to patients’ vision. Diabetes affects both anterior and posterior segments, which contribute to the gradual diminution of vision [1,2,3]. The retinal microvessels undergo several changes due to persistent hyperglycemia. These changes may be the development of microaneurysms, retinal hemorrhages, exudates, neovascularization, vitreous hemorrhages, tractional retinal detachment, and macular edema. They may be observed during fundus examination or angiography [4]. All of these hypoxia-induced microangiopathic changes are banded together as diabetic retinopathy.

Diabetic retinopathy (DR) is the most common cause of preventable vision impairment, mainly affecting the working-age group [5]. Though preventable, it is still the fifth leading cause of blindness worldwide [4]. The retinal changes usually appear after 5 to 10 years of disease onset. Initial non-proliferative stages of DR gradually progress to the proliferative stage in the presence of long-standing hyperglycemia. The timely diagnosis of diabetes, and strict glycemic control, halts the microvascular changes. However, reports suggest that diagnosis of diabetes is usually delayed by four to seven years [6]. By that time, pathologic changes might have already been initiated in a subset of the patient population, who must undergo screening tests for the detection of early changes.

Few available studies in the literature based on color Doppler ultrasonography (CDU) suggest that certain hemodynamic changes occur in the retrobulbar blood vessels, which precede the fundoscopic changes [7,8,9,10]. CDU is an established modality for the non-invasive radiation-free assessment of blood flow patterns in the orbital vessels. It allows for evaluation of blood flow through measurement of peak systolic velocity (PSV) and end-diastolic velocity (EDV), as well as indirect assessment of vascular resistance by calculation of resistive index (RI). The ocular blood vessels most frequently studied have been the ophthalmic artery, retinal artery, and posterior ciliary arteries. Few studies suggested a reduction in color flow in some, or all three, vessels [11,12,13], while others suggested an increase [14], or no change [15]. Similar disparities were observed while measuring the RI. Furthermore, it has also been suggested that these changes are more severe in the late stages of DR [16,17].

The Indian population, with more than 70 million diabetics, is highly prone to vascular complications of diabetes [18]. Therefore, it is imperative that the hemodynamic alterations responsible must be explored thoroughly, which could yield certain datasets which could be applied during the routine radiological assessment of orbital vasculature. In our study, we aimed to evaluate the retrobulbar arterial flow changes in diabetic patients with and without retinopathy, and to compare these changes with normoglycemic controls. The results from our study could provide a better insight into the vascular changes leading to, or occurring with, retinopathy.

## 2. Materials and Methods

A hospital-based cross-sectional study was carried out in our tertiary Maharaja Yeshwantrao Hospital between October 2020 and July 2021, after approval from the institutional review board.

### 2.1. Study Subjects

**Figure 1:**
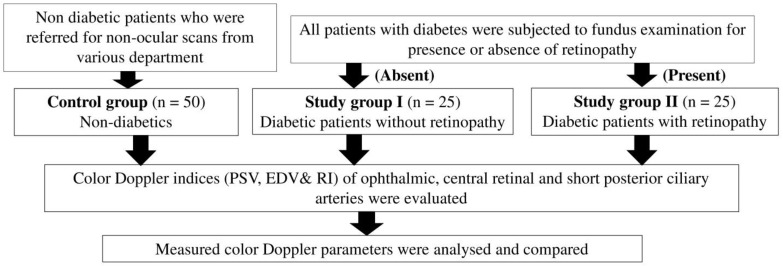
Flow chart showing study protocol. PSV—peak systolic velocity; EDV—end-diastolic velocity; RI—resistive index.

The study consisted of a total of 100 subjects, including 25 patients without diabetic retinopathy (Group I), 25 patients with retinopathy (Group II), and 50 non-diabetic controls (Figure 1). The diabetic status of the patients was assessed through documented hematological investigation for serum glucose levels/glycosylated hemoglobin. The patients who fulfilled the criteria, as per the guidelines of American Diabetes Association, were labeled as diabetics. All diabetics were then subjected to a detailed ocular examination. Dilated-pupil fundus examination was performed using a slit lamp with a 90 D lens and indirect ophthalmoscopy. Ophthalmoscopic findings suggestive of diabetic retinopathy are microaneurysms, retinal hemorrhages, retinal edema, hard exudates, cotton-wool spots, venous abnormalities (beading, looping, and dilatation), intraretinal microvascular abnormalities (IRMA), and dark-blot hemorrhages. Patients with such findings on ocular examination were labeled as Group II, while those without were banded together in Group I.

Patients who were referred to our department for non-ocular scans and who had normal blood glucose levels/normal percentage of glycosylated hemoglobin levels, were selected as controls. Informed consent, was obtained from both the cases and the controls before the commencement of the examination.

Exclusion criteria were patients with any anomaly or infection/inflammation of the eyes, those with a history of any previous ocular trauma/surgery/laser photocoagulation, smokers, patients suffering from glaucoma or systemic diseases such as hypertension, cardiovascular disease, non-diabetic vascular disease, nephropathy, etc., and those on medications that affect systemic circulation such as ACE inhibitors, CCB, and anti-migraine drugs.

### 2.2. Study Protocol

Demographic data and serum HbA1c levels were recorded for all participants. All 100 patients underwent CDU using an Esaote ultrasound system (model Mylab Seven) and a high-frequency (6–19 Megahertz) linear transducer, after written informed consent was given. CDU of the orbits was performed over closed eyelids with the patient in the supine position. The tip of the probe was covered with sufficient coupling gel to ensure adequate contact between the probe and the eyelids. Pressure to the globe was minimized by stabilizing the probe with the fingers on the forehead and cheek, to prevent patient discomfort and errors in Doppler parameters. The ophthalmic artery (OA) was scanned medial to the optic nerve. CDU-guided spectral samples were recorded from the central retinal artery (CRA) 5–10 mm posterior to the globe. Short posterior ciliary arteries (SPCAs) were evaluated on the temporal aspect along the periphery of the eyeball.

The peak systolic velocity (PSV) and end-diastolic velocity (EDV) were measured on the Doppler waveforms of all three vessels (Figure 1). Resistive index (RI) was calculated using the formula: RI = (PSV-EDV)/PSV. Color Doppler parameters of bilateral eyes were assessed for every patient, but the values from the worst eye were considered for analysis. To reduce errors in measurements, all ultrasonographic examinations were carried out by the same radiologist, and three measurements were taken for each Doppler parameter during the ocular ultrasound. The intra-observer reliability of the measured parameters was calculated using the intra-class correlation coefficient (ICC). The mean of these three measurements was documented. The obtained data were compiled, tabulated, and analyzed.

### 2.3. Statistical Analysis

Statistical Package for the Social Sciences (SPSS) software for Windows, Version 26.0. IBM Corporation (Armonk, NY, USA) was used for data analysis. The mean and standard deviations of the quantitative variables were calculated. A chi-squared test was used to compare the categorical variables. One-way ANOVA with post hoc Bonferroni was used to compare PSV, EDV, and RI among the three groups. Pearson’s correlation analysis was used to find an association between the status of the retina and the Doppler parameters. The receiver operator characteristic (ROC) curve was plotted to determine predictive values indicative of retinopathic changes. Two-tailed *p*-values of less than 0.05 were considered statistically significant.

## 3. Results

The study included 51 females and 49 males, out of which 29 females and 21 males belonged to the diabetic group. The age of the patients ranged from 45 to 65 (mean 51.8 ± 4.6) years in the diabetic group, and from 41 to 65 (mean 50.7 ± 6.1) years in the control group. The mean duration of diabetes in these patients was 7.3 ± 2.6 years, with a range of 3 to 13 years. The mean duration of diabetes was higher in those with retinopathy compared to those without (Table 1). The mean glycemic index (HbA1c) of the diabetic group was 9.1 ± 1.6, while that of the control group was 5.4 ± 0.3. The mean glycemic index was significantly higher in Group I compared to Group II (Table 1). Multiple measurements of the velocities and resistive indices for all three vessels (OA, CRA, and SPCA) were performed, and the intra-class correlation coefficient for the measured Doppler parameters lay between 0.75 and 0.82.

**Figure 2:**
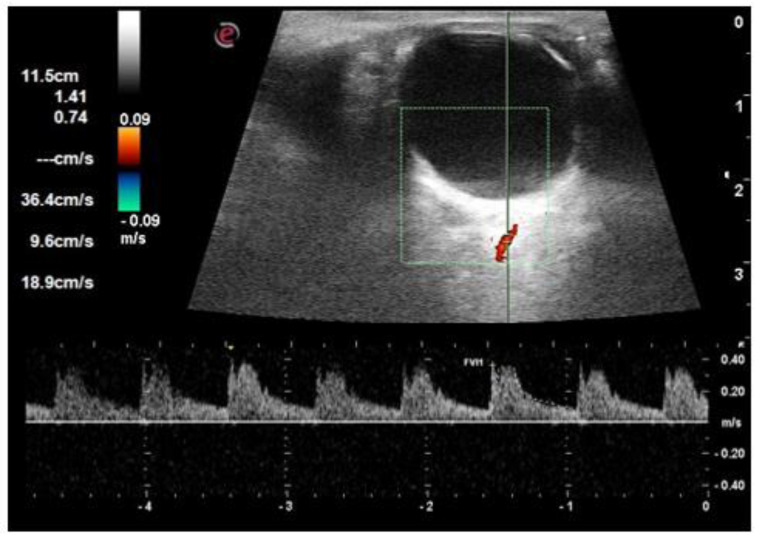
An orbital Doppler image of ophthalmic artery in a control subject shows a resistive index of 0.74.

**Figure 3:**
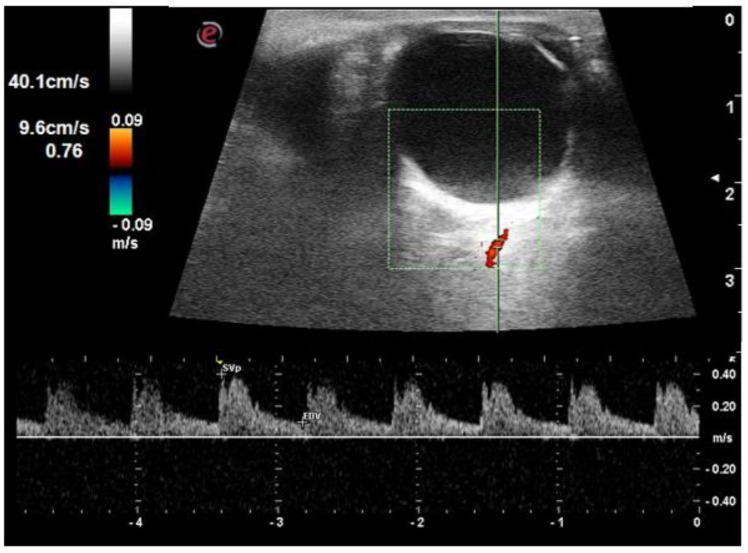
An orbital Doppler image in a patient with diabetes without retinopathy shows a resistive index of 0.76.

**Figure 4:**
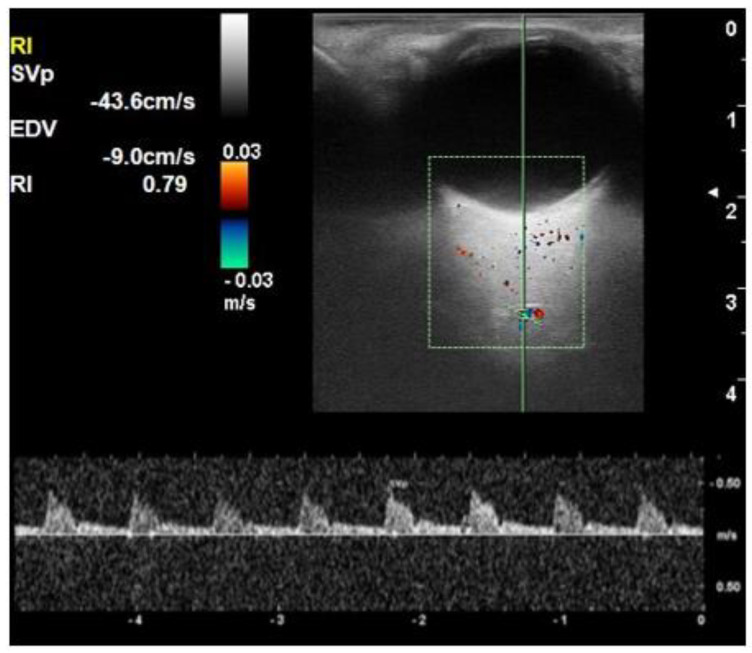
An orbital Doppler image in a patient with diabetic retinopathy shows a resistive index of 0.79.

**Figure 5:**
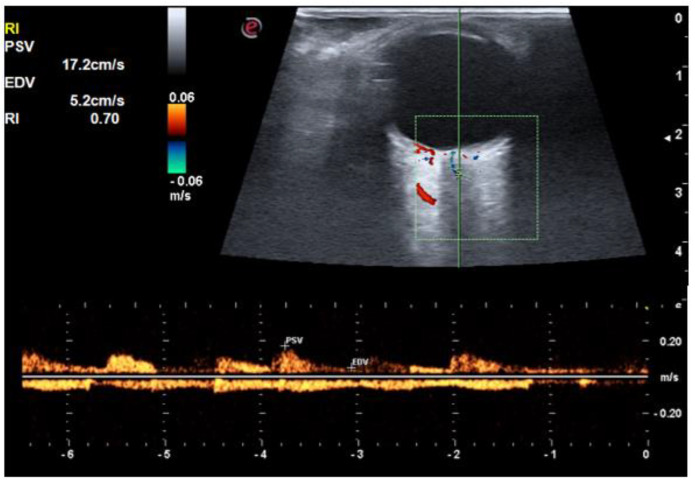
A color Doppler image of a diabetic patient without retinopathy shows a resistive index of 0.70 in central retinal artery.

**Figure 6:**
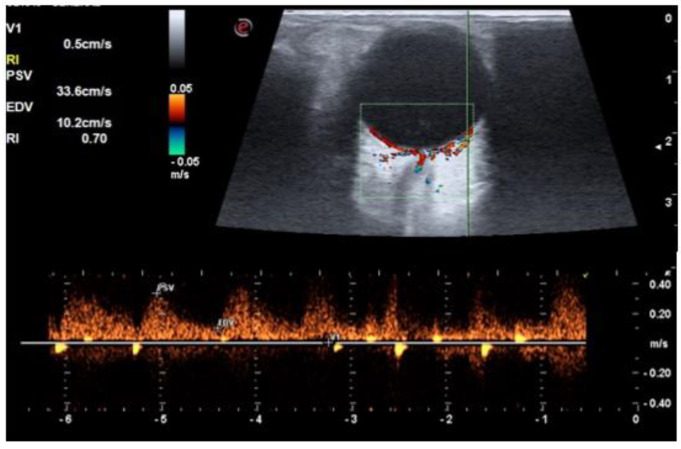
A color Doppler image ultrasound of a diabetic patient without retinopathy showing resistive index of 0.70 in short posterior ciliary arteries.

The RI of all three vessels (OA, CRA, and SPCA) was significantly higher in the diabetic group compared to the control group (*p* < 0.001) (Figure 2, Figure 3, Figure 4 and Figure 5). The difference was also significant between Group I and Group II for all three vessels (*p* = 0.004, *p* = 0.006, and *p* = 0.049, respectively). There was a significant reduction in PSV and EDV of CRA and SPCA in diabetic patients in comparison to normoglycemic patients (Table 1). The PSV and EDV of the ophthalmic artery were higher in diabetics compared to non-diabetics. However, this increase in Doppler parameters was not significant (*p* = 0.550 and *p* = 0.240, respectively). Subgroup analysis with post hoc comparison revealed significantly higher RI values in SPCA for Group I patients compared to the controls (*p* = 0.008) (Figure 6). Comparison of RI values of OA and CRA between Group I and controls showed no significant differences (*p* = 0.990 and *p* = 0.290, respectively).

**Figure 7:**
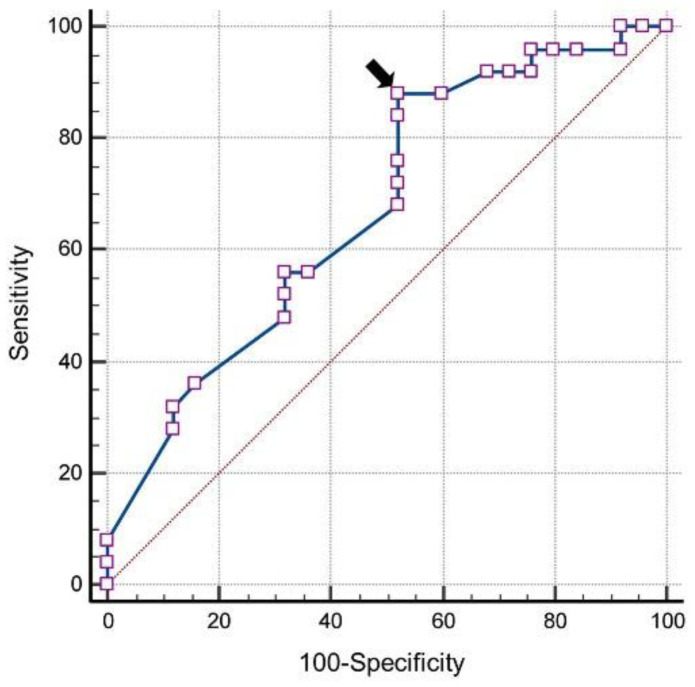
Receiver operating characteristic curve analysis of resistive index of the ophthalmic artery and diabetic retinopathy (the arrow shows the position of cut-off value).

**Figure 8:**
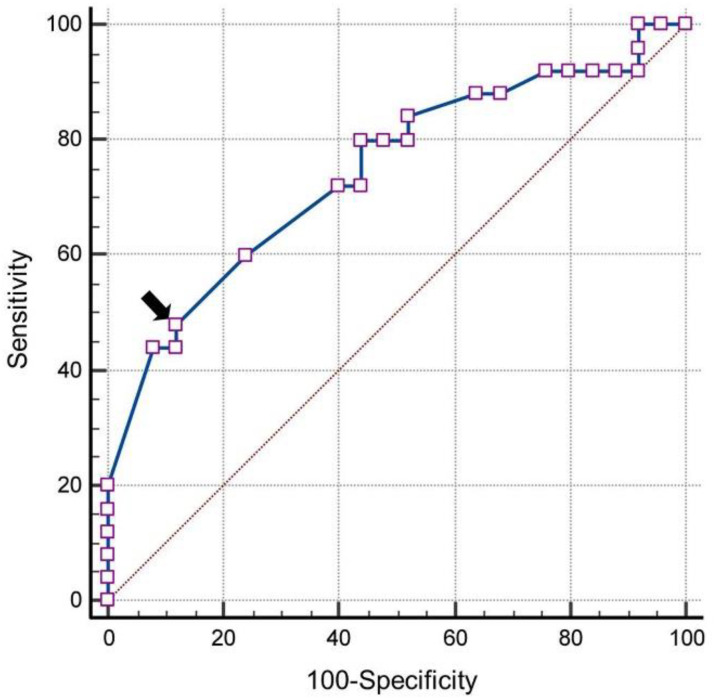
Receiver operating characteristic curve analysis of resistive index of the central retinal artery and diabetic retinopathy (the arrow shows the position of cut-off value).

Using ROC analysis, a cut-off of ≥0.73 for RI of OA yielded a sensitivity of 88% and specificity of 48% for the existence of retinopathy in diabetics (AUC = 0.667 (95% confidence interval: 0.516–0.818)) (Figure 7). RI of CRA ≥ 0.72 provided a sensitivity of 48% and specificity of 84% (AUC = 0.682 (95% confidence interval: 0.533–0.832)) (Figure 8). A Pearson correlation coefficient was computed to individually assess the linear relationship between RI of OA, CRA, and SPCA and presence of diabetic retinopathy. There was a positive correlation between the RI of retrobulbar vessels and DR (OA = r 0.417, *p* < 0.001; CRA = r 0.466, *p* < 0.001; SPCA = r 0.438 *p* < 0.001).

## 4. Discussion

DR is characterized by ocular vascular changes, which subsequently result in hemodynamic alterations. Retinal ischemia and subsequent hypoxia are thought to be the culprits causing the appearance of retinal lesions. However, there is conflicting evidence regarding the hemodynamic changes associated with retinal hypoxia [19,20]. CDU provides an indirect measurement of vessel blood flow [21,22]. In our study, we assessed the Doppler changes in retrobulbar vasculature of diabetic patients with retinopathy and compared them with the Doppler parameters in healthy controls.

Diabetes mellitus is characterized by increased resistance to blood flow due to increased blood viscosity, increased aggregation of RBCs and platelets, reduced compliance of RBCs while traversing microvessels, and platelet shape changes. Doppler study of flow in blood vessels provides an opportunity to assess the changes in vascular resistance through the measurement of RI. In our study, the RI of CRA and SPCA were significantly higher in patients with retinopathy compared to the controls. The increase in RI was accompanied by a decrease in the PSV and EDV of CRA and SPCA. The outer retina is supplied by choroidal vessels, while the inner layers are perfused by the central retinal artery. The reduction in blood flow during both the systolic and diastolic phases could be one of the triggers for hypoxia-induced retinopathy. There is a loss of capillary pericytes, thickening of the capillary basement membrane, increased stickiness of RBCs, and micro-occlusion in the retinal vasculature. The resultant capillary damage and dropout could be the reason for increased vascular resistance in CRA and SPCA. The hypoxia-induced capillary changes and capillary damage-induced increase in vascular resistance create a vicious cycle leading to progressive retinal changes.

Dimitrova et al. reported that the RIs of OA, CRA, and PCA are significantly increased in patients with diabetic retinopathy compared to control subjects [12]. Tamaki et al. also suggested that RI of OA is significantly higher in patients either with or without diabetic retinopathy than in normal subjects [9]. Basturk et al. showed higher RI values of the orbital arteries in patients with diabetic retinopathy than in patients without retinopathy [16]. All of these results are in concordance with the present study.

The hemodynamic changes with respect to PSV and EDV of OA were inconsistent in diabetic patients in comparison to controls. The OA, being a medium-sized muscular vessel, is unlikely to be influenced directly by the changes occurring in retinal capillaries. The CRA and SPCA, which lie in close proximity to the retina and are directly responsible for its nourishment, provide a better overview of the ongoing hyperglycemia-related pathologic changes. However, Doppler changes related to vascular resistance (RI) were also seen in the OA, and reflect downstream changes in its distal branches (CRA and SPCA).

A cut-off of ≥0.73 for RI of OA showed a sensitivity of 88% and specificity of 48%, and RI of CRA ≥ 0.72 showed a sensitivity of 48% and specificity of 84% in the prediction of DR. We found a positive correlation between RI values of retrobulbar vessels and the presence of DR among diabetes patients (Group I and Group II). All of these above results suggest a strong association between an increase in retrobulbar vascular resistance and the occurrence of DR. Therefore, RI is the best indicator among the studied Doppler parameters and can be used for screening diabetic patients.

The comparison of the flow parameters between Group I and the controls revealed a significant increase in RI of SPCA among diabetics without retinopathy. In these groups, there was no significant change in resistance of CRA and OA.

Therefore, we can hypothesize that changes denoting ischemia and insufficient perfusion are present long before the overt fundal changes. Several previous studies based on laser Doppler flowmetry, optical coherence tomography, and CDU have shown decreased choroidal blood flow in the early stages of diabetic retinopathy [23,24,25], which fortifies our proposed hypothesis. The changes observed also suggest that choroidal vessels are more appropriate for early detection of diabetes-associated retrobulbar flow changes.

Our study excluded all hypertensive patients and patients taking drugs that alter vessel hemodynamics. Therefore, the vascular changes observed in our study strongly represented diabetic etiology. The correlation between ophthalmoscopic and CDU findings in all three groups involved in our study has provided a better insight into the vascular changes. Our study represents one of the few comparative studies performed using CDU, especially among the Indian diabetic population. However, there are certain limitations to our study. Our study was a cross-sectional study, which failed to serially assess the Doppler flow changes among diabetics, which would have provided a better analysis of the disease process. Due to the limited sample size, we could not study and correlate the hemodynamic changes with the sub-categories of diabetic retinopathy—mild, moderate, and severe non-proliferative, and proliferative diabetic retinopathy. Although the CDU was carried out by the same radiologist in our study with low intra-observer variability, small variations in measurements with different cut-off values can substantially change the sensitivity and specificity values. Future cohort studies with larger sample sizes are required for a better understanding of the variation in orbital CDU parameters. Furthermore, all the measurements in our study were taken in a single sitting. Multiple measurements of the same subject at different time intervals may help us understand whether the results are reproducible for screening or follow-ups.

## 5. Conclusions

Significant changes occur in retrobulbar circulation in diabetic patients, especially those with retinopathy. RI of OA, CRA, and SPCA can potentially be used for the diagnosis of DR. This is of particularly great importance in patients where visualization of the fundus is difficult due to the opacification of structures anterior to the retina.

## Figures and Tables

**Table 1:** Characteristics of subjects in control and study groups.

Characteristics	Control (*n* = 50)	Group I (*n* = 25)	Group II (*n* = 25)	*p*-Value
Age (years)	50.7 ± 6.1	50.8 ± 4.3	53.1 ± 4.7	0.177
Duration of diabetes (years)		6.2 ± 2.5	8.4 ± 2.3	0.002
HbA1c (%)	5.4 ± 0.3	8.4 ± 1.2	9.9 ± 1.6	0.001
PSV of ophthalmic artery (cm/s)	31.9 ± 2.6	32.7 ± 3.0	33.8 ± 3.8	0.550
EDV of ophthalmic artery (cm/s)	8.7 ± 0.9	8.8 ± 1.6	8.3 ± 1.3	0.240
RI of ophthalmic artery	0.72 ± 0.02	0.73 ± 0.03	0.75 ± 0.03	<0.001
PSV of central retinal artery (cm/s)	16.1 ± 1.9	15.8 ± 3.1	14.2 ± 2.7	0.010
EDV of central retinal artery (cm/s)	5.1 ± 0.7	4.8 ± 1.1	3.9 ± 1.0	<0.001
RI of central retinal artery	0.68 ± 0.03	0.69 ± 0.03	0.72 ± 0.04	<0.001
PSV short posterior ciliary arteries (cm/s)	19.1 ± 2.5	18.0 ± 4.2	16.7 ± 4.9	0.030
EDV short posterior ciliary arteries (cm/s)	5.9 ± 0.7	5.1 ± 1.3	4.3 ± 1.0	<0.001
RI short posterior ciliary arteries	0.69 ± 0.03	0.71 ± 0.03	0.73 ± 0.5	<0.001

Group I—diabetic patients without retinopathy; Group II—diabetic patients with retinopathy. PSV—peak systolic velocity; EDV—end-diastolic velocity; RI—resistive index.

## Data Availability

The data are not publicly available to maintain the privacy of study subjects.
